# The impact of stigma on HIV testing decisions for gay, bisexual, queer and other men who have sex with men: a qualitative study

**DOI:** 10.1186/s12889-022-12761-5

**Published:** 2022-03-09

**Authors:** Bradley E. Iott, Jimena Loveluck, Akilah Benton, Leon Golson, Erin Kahle, Jason Lam, José A. Bauermeister, Tiffany C. Veinot

**Affiliations:** 1 School of Information, University of Michigan, Ann Arbor, MI USA; 2 Department of Health Management and Policy, School of Public Health, University of Michigan, Ann Arbor, MI USA; 3 Washtenaw County Health Department, Ypsilanti, MI USA; 4 Detroit Health Department, City of Detroit, Detroit, MI USA; 5 Unified – HIV Health and Beyond, Ypsilanti, MI USA; 6 School of Nursing, University of Michigan, Ann Arbor, MI USA; 7 School of Nursing, University of Pennsylvania, Philadelphia, PA USA; 8 Department of Health Behavior and Health Education, School of Public Health, University of Michigan, Ann Arbor, MI USA

**Keywords:** Stigma, HIV testing, Decision-making, Men who have sex with men, Detroit

## Abstract

**Background:**

Stigmatization may prompt gay, bisexual, queer and other men who have sex with men (GBQMSM) to avoid or delay HIV testing. There has been little attention to GBQMSMs’ perspectives about *how* stigma may influence their decisions about whether, where, and how often to get tested for HIV.

**Methods:**

We conducted nine focus groups with 64 adult GBQMSM in Metropolitan Detroit, including HIV-negative men and people living with HIV (PLWH). Data were thematically analyzed deductively and inductively in three rounds.

**Results:**

Three themes emerged regarding *whether to get tested*: (1) Perceived promiscuity, risk perceptions and HIV testing; (2) Fearing sexual rejection; and (3) Fearing friend and family member distancing and rejection. Themes concerning *where to get tested* included: (4) Conflating HIV testing and diagnosis; and (5) Seeking privacy and safety at specialized services. As for *how often to get tested*, themes included: (6) Reducing contact with healthcare providers due to intersectional stigma; (7) Responsibility and regular testing; and (8) HIV stigma and testing as routine care. Black participants articulated themes (3), (4), and (6) with greater frequency than other participants. Framing HIV testing as a personal responsibility may have created a “new stigma,” with unintended consequences not observed with “routine healthcare” messaging.

**Conclusions:**

GBQMSMs’ perspectives indicate the potential for new foci for HIV testing promotion interventions based on stigma-related issues that they deem important. There is a need for interventions to challenge the “promiscuity” stereotype, and to reduce the sexual stigmatization of GBQMSM living with HIV/AIDS—especially online. Provider stigma requires both intervention and continued availability of specialized services. Future stigma-reduction interventions for Black GBQMSM could focus on building family support/acceptance, awareness of multiple testing options, and integrating LGBTQ-related issues into initiatives for racial justice in health care.

**Supplementary Information:**

The online version contains supplementary material available at 10.1186/s12889-022-12761-5.

## Background

Gay, bisexual, queer and other men who have sex with men (GBQMSM) in the United States (US) are disproportionally affected by HIV; indeed, 69% of new HIV diagnoses in 2018 were among GBQMSM [1]. In Metropolitan Detroit, GBQMSM make up more than half of all reported HIV infections and Black GBQMSM experience a disproportionate share of this burden [2]. HIV status awareness, which requires regular testing, may expand uptake of prevention options such as pre-exposure prophylaxis (PrEP), and facilitate early entry into care and treatment as prevention (TasP) for people living with HIV (PLWH). Thus, significant policy and program efforts aim to expand testing behavior among GBQMSM.

However, HIV-related stigma—a social process characterized by the co-occurrence of labeling, stereotyping, separation, status loss, and discrimination [3]—is a barrier to HIV testing [4–14]. Public HIV-related stigma remains prevalent in the US population; a recent survey using a US national probability sample found that 17.5% of adults and 31.6% of adolescents feared casual contact with people with HIV/AIDS, and 12.5% expressed moral judgement towards them [15]. Moreover, 21% of GBQMSM in a recent survey believed that “most people” would discriminate against PLWH—with little change in the prevalence of this perception over time [16].

This public stigma influences individuals’ feelings about, and perceptions of, the social environment. Results include fears of stigma [4, 12, 13, 17–19], and cognitive expectations of negative consequences of living with HIV/AIDS, including negative treatment (“anticipated stigma”) [5, 6, 20, 21]. In turn, stigma-related fears and expectations are associated with delays in, and infrequent, HIV testing [5, 6, 17, 18, 21]—or it may be linked to non-testing [4, 12, 13] and testing refusal [14].

Importantly, GBQMSM who seek testing face stigma related not only to a possible HIV diagnosis, but also to same-sex sexual behaviors [14]. Moreover, HIV-related stigma itself is intertwined with negative attitudes, often expressed interpersonally between people, towards same-sex sexual practices and identities (e.g., [22]). Reflecting this intertwinement, a recent survey of Australian GBQMSM found that 70% reported experiencing interpersonal stigma due to perceived HIV risk despite the fact that only 5% actually reported being that they were PLWH [23]. Perceptions of stigma towards GBQMSM may also interact with psychological distress to reduce HIV testing behavior [24].

Furthermore, GBQMSM living with HIV experience “intersectional stigma,” or the convergence of multiple stigmatized identities [25, 26], in relation to both their sexual identities/practices and disease status. GBQMSM of color may also face racism as an intersecting stigma along with homophobia, and this intersection has been linked to HIV testing behavior [13, 27]. Transgender and/or gender-nonconforming GBQMSM may also face transphobia and gender non-affirmation; gender non-affirmation from cisgender male partners has been associated with decreased odds of HIV testing among transgender GBQMSM [28]. Notably, men who have sex with both men and women also face unique stressors, such as biphobia [29, 30], when compared to men who only have sex with men. Moreover, high levels of internalized homonegativity may serve as a barrier to HIV testing among behaviorally bisexual men [31]. Men who have sex with men and women are more likely to have delayed HIV diagnosis than men who only have sex with other men [32].

These forms of stigma—anticipated, interpersonal and intersectional—are all linked to “structural stigma,” or “societal-level conditions, cultural norms, and institutional policies that constrain the opportunities, resources, and wellbeing of the stigmatized” [33]. Structural stigma, which may emerge both intentionally or as an unintended consequence of other policies [34], and which has roots in multiple social institutions [35], has been associated with HIV-related behaviors and outcomes for GBQMSM [36–40]. Structural stigma may specifically manifest in “provider-based stigma” [26], or “prejudice and discrimination voiced or exercised, consciously or unconsciously, by occupational groups designated to provide assistance to stigmatized groups” [41]. Furthermore, stigma may also be internalized as negative feelings about one’s own identity (“intrapersonal stigma” or “self-stigma”) [42], which may also influence GBQMSMs’ HIV testing behavior [43].

While the aforementioned research has documented the importance of multiple forms of stigma for GBQMSM in general and HIV testing behavior in particular, there is a need to better explain the processes by which different forms of stigma affects individuals [44], including their health-related decisions. Moreover, despite extensive recent attention to stigma’s negative impact upon GBQMSMs’ decisions to take PrEP [45–49], there has been less attention to HIV testing, which is critical given its link to PrEP and TaSP. To understand how stigma influences HIV testing, it is important to investigate its impact on several micro-decisions that comprise testing behavior. These include deciding: (1) whether to get tested [50]; and (2) where to get tested [50–54]. Beyond an individual testing episode, frequency of testing is also an important micro-decision [50] given CDC recommendations that sexually active GBQMSM receive an HIV test at least annually. This directive is frequently not met among GBQMSM in the US (e.g., [55, 56]). Critically, some research shows that different testing micro-decisions may be influenced differently by stigma [7]—suggesting a need for further research in this area.

Additionally, despite numerous survey-based studies on HIV testing correlates [5, 6, 17, 18, 21], and qualitative research about testing barriers and facilitators in different populations (e.g., [57]), there has been relatively little in-depth attention to GBQMSMs’ perceptions of how stigma affects each of their HIV testing micro-decisions. Yet, there is a recognized need for more research to “understand and address the challenges and needs of the stigmatized” [58]. In an HIV/AIDS and GBQMSM context, qualitative research is particularly valuable for such research because it facilitates understanding of: the contextually-embedded nature of stigma [41], interactions between levels of stigma [59], and ways in which stigma-related contextual factors and social processes may influence health decision making [60]. It is also helpful for developing stigma reduction interventions by providing “nuanced understanding of stigma in a given context from the perspective of those to be targeted by stigma reduction efforts” [59]. Accordingly, we used qualitative methods to pursue study objectives.

### Study Objectives

To identify GBQMSMs’ perspectives regarding how stigma (interpersonal, intersectional, structural, provider-based, self-stigma) influences their HIV testing decisions, including micro-decisions regarding whether, where, and how often to get tested.

## Methods

### Sample

 We conducted a community-based participatory research study involving Unified - HIV Health and Beyond (Unified), an HIV/AIDS service organization (ASO) in Metropolitan Detroit; this ongoing partnership began in 2010. In line with qualitative sampling approaches [61], purposeful sampling was used to recruit participants with varied relationships to HIV testing-related stigma (those who have tested positive for HIV and those who have not, GBQMSM who experience intersecting stigmas such as racism) and with different methods of engaging with their local communities (e.g., dating applications, social media, email groups, and in restaurants, bars, clubs, university buildings, LGBTQ community center, ASO) since this may reflect differences in underlying social networks and attitude exposure (e.g., [62]). As described in [63], potential participants were approached with social media ads, posted paper flyers, emails, and through interpersonal contact via phone or email. Participants in the PLWH focus groups had prior relationships with UHHB staff; many had participated in UHHB programs in the past. Eligible participants were self-identifying men who had sex with men in the past six months (see Table 1 for their reported sexual identities) and lived in Metropolitan Detroit. This included cisgender (n=61) and transgender or gender-nonconforming men (n=3).

**Table 1:** Characteristics of Participants

	Focus Group Participants (n=64)	
**Characteristic**	**Number / Mean (SD)**	**Percentage**
**Age (Mean) (SD)**	38.6 (14.3)	
**Race/Ethnicity**		
Black or African American	30	46.9%
White	29	45.3%
Hispanic/Latino	5	7.8%
Asian	4	6.3%
American Indian or Alaskan Native	0	0%
Other	1	1.6%
**Sexual Identity**		
Gay	51	79.7%
Bisexual	10	15.6%
Other	3 – “Queer”	4.7%
**Ever Tested for HIV**		
Yes	57	89.1%
No	2	3.1%
No Answer	5	7.8%
**Number of times tested for HIV (Mean) (SD)**	6.94 (9.6)	-
**HIV Status (Self-Reported)**		
HIV-Positive	31	48.4%
HIV-Negative or Status Unknown	33	51.6%
**Years since HIV diagnosis**	12.92 (9.92)	

### Data Collection

We collected data through nine, one-time face-to-face focus groups with 64 adult GBQMSM in 2016-2017 (two hours on average). To facilitate sharing experiences as a potential form of empowerment [59], and to reveal group meanings and norms [64], and dynamics underlying discussion of stigma [65], we used focus group discussions to collect data. Focus groups used a discussion guide developed in draft form by TCV, followed by revision based on feedback from Unified Staff (See Supplementary materials), and centered on experiences of HIV testing and prevention, stigmatization, social networks, and community dynamics in Metropolitan Detroit. Although GBQMSM who were PLWH were present in all focus groups, in order to promote greater comfort in discussing sensitive experiences, three were specifically for them. Focus groups were led by a both Unified staff (AB, Female, then Community Mobilization and Research Manager and LG, Male, Director of Prevention Programs) and University of Michigan researchers trained in public health, health informatics and qualitative methods (TV, Female, Professor and BI, Male, PhD Student). Focus groups were held in private, closed-door rooms at two different UHHB offices, in an LGBTQ+ community center, and on the University of Michigan campus. No one other than the researchers and participants were in the room. Facilitators were introduced as researchers from the University of Michigan who were working with UHHB on projects to help inform intervention development. In addition to handwritten notes, focus groups were audio recorded, professionally transcribed, and verified by UM staff. Transcripts were shared with Unified staff, but not focus group participants. Recruitment continued until data saturation was reached, such that no new empirical findings were emerging by the final focus group [66]. All provided informed consent, and participants knew the goals of the research. The study was approved by the University of Michigan Institutional Review Board.

### Analytic Strategy

Using a thematic analysis approach [67], three authors coded focus group transcripts in three rounds using NVivo software. The first round involved inductive, open coding [67] that focused on sources of stigma and stated links between stigma and testing and deductive codes drawing from prior literature concerning stigma (e.g., codes for Link and Phelan’s stigma definition, including “labeling,” “stereotypes,” and “social distancing” [3]) and literature on HIV testing micro-decisions (e.g., “where to seek testing,” and “how often to get tested.”) The second coding round involved process coding and in vivo coding to hone in dynamics of stigma’s relationship to HIV testing decisions [3]. A third round of selective coding was completed to subsume dominant codes into themes [38]. University of Michigan staff met regularly with UHHB representatives to verify the codebook and evaluate evidence supporting codes [39]. For the purposes of contextualization while maintaining participant confidentiality, individual years since diagnosis are reported in quartiles (Q1: 0-4.50; Q2: 4.51-10.99; Q3: 11-21.99; Q4: 22-31).

## Results

### Characteristics of Participants

Most participants (79.7%) identified as gay. Their average age was 38.6 years. Black and White GBQMSM were the largest racial groups, followed by Hispanic/Latino GBQMSM (Table 1). Nearly half (48.4%) were living with HIV, and their average years since diagnosis was 12.92 (range: 0-31 years). Overall, 89.1% had ever had an HIV test, with a mean of 6.94 tests per participant.

### Findings by HIV testing decision

The findings presented below outline themes regarding ways in which stigma affects three HIV testing decisions: (a) whether to get tested, (b) where to get tested, and (c) how often to get tested.

#### Whether to get tested

“They had a slutty evening”: Perceived promiscuity, risk perceptions and HIV testing.

Some GBQMSM felt that testing, including repeatedly, would lead to stereotyping as sexually promiscuous—a stereotype applied to GBQMSM and judged as immoral. As this man said,

*“If someone gets tested…frequently, then someone would assume that they are sexually promiscuous. The stigma is also there for that.”* (FG8-1, White, Gay, 32, HIV-negative).

It was felt that this could make decisions to test less likely. Relatedly, GBQMSM expressed beliefs that testing was only necessary after “slutty” behavior. For example, a participant claimed that people get tested because *“They had a slutty evening, truly.”* (FG1-7, Black, Gay, 37, PLWH, 4.51-10.99 years since diagnosis) Similarly, identifying one’s behavior as promiscuous could prompt perceptions of being at risk and in need of HIV testing,

*“I’m a ho. Card-carrying. And I’m a realist, okay? The last two guys I was with, I think they kinda cool but I have one… I think it’s questionable and I gotta get my ass up here and get tested. See what’s really goin’ on.”* (FG3-12, Black, Gay, 64, HIV-negative).

However, concerns about promiscuity and HIV risk could also spawn fear and HIV test avoidance, as this participant explained,

*“…this one period where I just refused to get tested…because I knew that I had been having way too much sex. It’s was like, ‘If I get a positive results back, I won’t know what’s gonna happen.’”* (FG3-4, Black, Gay, 24, HIV-negative).

At the same time, not seeing oneself as promiscuous could facilitate inaccurate risk perceptions, and delayed HIV testing, as this participant who had tested only for an incentive explained,

*“I thought I was doing everything right… I was like, I’m not a… I’m a wild girl, but [laughter]…I’ve been around but not* ***around***…*”* (FG2-7, Black, Gay, Age not given, PLWH, Years since diagnosis not given).

Multiple participants commented on moral judgement about perceived promiscuous behavior. Fear of such judgements from healthcare providers could further stand in the way of testing,

*“It may be very hard to go in and get that test when you know you had sex with multiple people, unprotected, you know, ‘Here I go again.’”* FG3-5 (Asian, Gay, 22, HIV-negative).

There was also acknowledgement that GBQMSM judged one another for perceived promiscuity, although opinions were mixed as to how pervasive or damaging this was. One joked that the GBQMSM community was divided into, *“Those that admit to whoring around and those that don’t in particular.”* (FG4-1, Latino, Bisexual, 45, HIV-negative) However, other men felt that stigma about “sluttiness” was a concern. A few men stated that this was a form of self-stigma. For example,

*“…in my experience, gay men are…more judgmental about how many partners one has… it comes back to internalized homophobia… to deviation from the normal married with 2.2 kids standard, that you’re not following the rules of the society…”* (FG8-3, White, Gay, 38, HIV-negative).

GBQMSM who were PLWH also mentioned the potentially damaging nature of the promiscuity and HIV stereotype to them personally, *“I feel like I’m constantly being persecuted…People think it’s just, “Oh, you’re being loose.“ I was in a committed relationship.”* (FG2-5, Black, Gay, 44, PLWH, Years since diagnosis not given) Such stigma, when expressed towards PLWH, may subsequently influence HIV-negative GBQMSM as well.

“When you’re online talking to someone, the first thing that comes up, ‘Are you clean?’”: Fearing sexual rejection.

Many participants, but particularly those under 30 years of age, highlighted fear of sexual rejection as a barrier to deciding to seek HIV testing. It was felt that having HIV made someone a “less desirable” sexual partner, which might undermine one’s ability to form relationships or result in being ignored or neglected. According to this man, this could be particularly difficult for GBQMSM who already had difficulty finding partners, *“…If they’re someone who’s already low…‘Oh I have a hard enough time getting a guy, now I just gotta add HIV to it’ they’re not gonna [seek HIV testing].”* (FG2-3, Black, Gay, 22, PLWH, 0-4.50 years since diagnosis).

 Several participants stressed that the responsibility to disclose an HIV diagnosis would being emotionally challenging and undermine burgeoning sexual encounters,

*“They’re afraid that if they find out that they’ll be stigmatized and then they have a duty at that point to tell people…and then if you do wanna go out and have hook-ups, it’s kind of a deflating statement if you went and said to somebody right before sex that ‘Oh, I’m HIV positive.’”* (FG4-3, White, Gay, 29, HIV-negative).

Participants’ sensitivities to the possibilities of sexual rejection were acute after having witnessed stigmatizing online interactions concerning HIV—especially on hookup apps such as Grindr and Scruff. Although most rejected the practice themselves, many participants highlighted the prevalence of online profiles that stated an interest only in HIV-negative men on such sites. As this man said,

*“…I’ve also seen the kind of social stigma around [HIV] impact people to be fearful of even being tested, but the fear of knowing… I’ve seen people be shunned [on hookup apps] because of it…”* (FG9-8, White, Gay, 27, PLWH, 0-4.50 years since diagnosis).

A participant, however, admitted to personally rejecting potential partners on the basis of HIV status,

*“…on…dating apps…“…if you see an HIV positive tag on someone’s profile, I know a lot of people myself included, just ignore that person. I think a lot of people are just afraid of getting HIV and don’t want to take any sort of risk, and find it easier to just kind of ignore that person than to take other types of precautions and still give the person a chance.”* (FG7-7, White, Gay, 25, HIV-negative).

However, participants noted a counter-trend towards resisting stigma on the same sites,

*“I think you see that a lot on Grindr profiles and Scruff profiles, where it’s some people are very affirming. They’ll put the equal sign within their name. They don’t sort by their status but other times you see people say ‘clean only.’”* (FG8-4, White, Gay, 23, HIV-negative).

Participants also complained about the ubiquitous use of the term “clean” to designate “HIV-positive status” when negotiating sexual encounters. Typically, participants perceived this question as an effort to quickly sort “HIV-positive” from “HIV-negative.” One said,*“It’s jarring when you’re online talking to someone, and the first thing that comes up, ‘Are you clean?’”* (FG6-2, Black, Gay, 56, HIV-negative). GBQMSM who were living with HIV found cleanliness metaphors common, painful, and demeaning. A few men also expressed disappointment that they were not privy to more substantive HIV-related discussions than *“are you clean?”* in the community.

 Additionally, a few participants experienced social judgement due to relationships or sexual encounters with PLWH, which further shaped their perceptions of the potential for sexual rejection. For example, a participant was surprised when a friend dated a positive man, and another said,

*“I’ve had people after finding out that I have slept with people that I knew were positive, like, ‘Why would you do that?’… I perceive the stigma…as [a] higher barrier than the fear of being positive…having to say that when negotiating sex or… put that on your profile.’”* (FG7-8, White, 26, Queer, HIV-negative). However, a few expressed the belief that PrEP and TasP had begun to soften some of the HIV-related divisions between GBQMSM. Yet several cited mandatory disclosure laws, which were in place in Michigan at the time of the study, as disincentivizing testing,

*“I think our current legal system with the mandatory disclosure laws and everything, it does not encourage testing because it basically is like, ‘If you don’t know, you’re fine.’”* (FG3-6, Asian, Gay, 21, HIV-negative).

“I’m not gonna have that happen to me and my family”: Fearing friend and family member distancing and rejection.

 According to participants, the possibility of rejection from friends or family members could provide a barrier to the decision to seek HIV testing. Particularly a concern among Black and older White participants, there was a feeling that people could lose valued social relationships as a result of an HIV diagnosis. A man said,

*“I probably would have never [gotten tested] until my guy I was talking to told me to do it, and we went together. Because I would’ve thought that if I was positive I was gonna lose my family…”* (FG1-8, Black, Gay, 45, PLWH, 4.51-10.99 years since diagnosis).

In addition to fears grounded in one’s personal family relationships, participants who were PLWH felt that others hearing “horror stories” about their experiences could deter them from testing,

*“S: Falling out with my family, right? Right, so if I’m with somebody and they haven’t had that experience, and they hear me talking, they’re like, ‘Oh no, fuck that. I’m not gonna have that happen to me and my family, because I wanna be close to them.’”* (FG1-7, Black, Gay, 37, PLWH, 4.51-10.99 years since diagnosis).

Another participant was deeply affected by the stigma that he had witnessed early in the epidemic, and he had avoided testing out of fear for many years,

*“[I] remember the bad old days…That was really when people were sick and shunned…People are afraid to know their status…They’re afraid to be stigmatized by people finding out that they’re HIV positive…myself, when I was younger, [fear of stigma] did [affect my HIV testing decisions].”* (FG8-3, White, Gay, 38, HIV-negative).

Relatedly, participants spoke of negative experiences in which family members or friends attempted to distance themselves due to perceived contagion after they received a positive HIV test. Events included wearing gloves at a participant’s house, wanting separate toothpaste, a request to not bring food to a family cookout, anxiety about accidentally sharing water, and anger at someone holding a child. While most of these events had taken place in the past, some were recent. Regardless of timing, these forms of enacted stigma remained distressing for PLWH participants, and for them, remained a factor making testing more difficult for others,

*“…[friends] don’t wanna talk to you anymore, they don’t want you to be around their kids. They’re just not sure what to do…so you lose people…You have to start all over again almost, especially if you don’t have a supportive family.”* (FG3-1, Black/Latino, Gay, 53, PLWH, 22-31 years since diagnosis).

#### Where to get tested

“Guilty until proven innocent”: Conflating HIV testing and diagnosis.

 Many Black participants expressed concerns about being labeled “HIV-positive” by others if others learned of their HIV testing behavior. Here, the mere association of one’s name or identity with testing was enough for others to assume they were living with HIV,

*“All you have to do is say, “I’m going to go get a HIV test.“ Right? Whether it’s positive or negative, you’ve already identified yourself with HIV. So, it’s an assumption that that is your diagnosis, whether it is or not.*” (FG1-1, Black, Gay, Age not given, PLWH, Years since diagnosis not given).

Furthermore, this labeling of testers as positive could be long lasting, *“…if you say something, it just sticks… they don’t hear, ‘I’m gonna get tested for HIV…’ They hear, ‘HIV’…* (FG3-1, Black/Latino, Gay, 53, PLWH, 22-31 years since diagnosis) Participants felt that others might assume they were living with HIV, either if they disclosed testing behavior, or if they were seen at an HIV testing location, especially within smaller communities *“I’m constantly running to people I know. So in this area, who wants to be seen getting a test or be associated…?”* (FG1-3, Black, Gay, 25, PLWH, 4.51-10.99 years since diagnosis).

Several participants felt that although a man’s HIV test might be negative, he might still face social consequences due to testing, such as rejection or being the subject of gossip, especially, *“when everybody basically knows everyone.”* (FG6-5, Black, Gay, 33, PLWH, 11-21.99 years since diagnosis). A few participants further expressed concern about the potential for gossip about their HIV status. As one said,

*“…the main issues was in the black community, the ballroom scene,…you’re guilty until proven innocent. If I walk into an STD clinic and I know that they test for HIV here, I see you here, I’m already gonna think, “Oh, you’re positive. Let me go tell everybody.”* (FG6-5, Black, Gay, 33, PLWH, 11-21.99 years since diagnosis).

This primarily led to concerns about privacy during testing, particularly when in clinic waiting rooms or seeking services within one’s community. A participant explained,

*“I’m in Detroit, I’m a black male, right? They have these black organizations all over Detroit. Oh, hell no. I went to the Hispanic organization down in Southwest Detroit, and if there was a blizzard or a thunderstorm, they could expect me as a walk in, okay? I will be there… I wanted to be in there with nobody else.”* (FG3-12, Black, Gay, 65, HIV-negative).

*“I refuse to get tested anywhere else”: Seeking privacy and safety at specialized services*.

Many participants sought HIV testing from services that they believed would spare them negative experiences of stigma about their sexual behavior—including AIDS Service Organizations (ASOs), community health centers or GBQMSM-competent physicians. Some of these participants described experiencing stigma from healthcare providers, whether in relation to testing, PrEP or PEP care that resulted in decisions to seek testing in different locations in the future. For example, one said,

*“I went to go get a PEP [Post-Exposure Prophylaxis] after a really bad experience…Was turned away from two ERs [Emergency Rooms]. One doctor was incredibly homophobic. I was bawling…[now] I refuse to get tested anywhere else besides [LGBT nonprofit that partners with county public health] or [ASO]”* (FG5-4, White, Queer, 26, HIV-negative).

Another participant reported denial of care from a clinician after he asked him numerous invasive questions,

*“I had a family practice nurse practitioner, unfortunately, deny me STI testing and prescribing PrEP and actually told me to go to Planned Parenthood… There still is a lot of stigma and that was probably the most slap in the face I had ever had…”* (FG8-1, Gay, White, 32, HIV-negative).

In addition to care denial, some participants reported unpleasant reactions from clinicians when they discussed their HIV prevention needs. One man explained,

*“I had a doctor who I said I want to have HIV testing and he looked at me like, ‘You’re gay?’ It was like… So that would be a hindrance. Of course, I also switched doctors… I went to a gay doctor.”* (FG4-2, Gay, White, 49, HIV-negative).

As suggested above, such experiences led to decisions to change providers to those thought to be more competent in GBQMSM care. However, concerns about the competence of physicians also intersected with issues related to insurance access; participants felt that having good health insurance increased the odds of having good experiences. As this participant said,

*“I got really good healthcare. So I got tested in January from my doctor…I have access to get my blood taken from the lab at my doctors so I think it’s all about access.”* (FG9-6, White, Gay, 45, HIV-negative).

However, younger men insured under their parents’ plans feared their parents learning about their HIV testing behavior. This man whose last test was through an LGBTQ community center said,

*“As a dependent of parents who don’t know that I’m gay, I think having a HIV test on your insurance statement can really cause some problems. And…I’ve had some really bad experiences with doctors that are not very LGBTQ friendly….”* (FG3-6, Asian, Gay, 21, HIV-negative).

In fact, some study participants said that they had experienced unwanted parental disclosures of their sexual health service use due to relying on parents’ insurance and payment for care. Other insurance concerns related to employment and possible insurance discrimination, which resulted in seeking anonymous testing,

*“I would always…get tested anonymously because I didn’t want my insurance company to know…If I had seroconverted, hi, preexisting condition. ACA doesn’t exist yet. I am screwed.”* (FG7-4, White, Gay, 48, HIV-negative).

#### How often to get tested

Among participants, there was general acknowledgement that GBQMSM who were not in monogamous relationships should be tested more than once, or “regularly.” As this man said, *“If you’re not monogamous, sexually active gay man, it’s just responsible to get tested*

*regularly.”* (FG3-9, White, Gay, 33, HIV-negative) However, participants believed that stigma stimulated deviation from this ideal; at the same time, stigma about *non-*testing supported frequent testing in some GBQMSM subgroups.

“People have a fear of health systems and doctors”: Reducing contact with healthcare providers due to intersectional stigma.

Black GBQMSM expressed opinions that HIV testing might occur less frequently than recommended due to histories of, and direct experiences of, racism as a form of intersectional stigma. As one said, *“…there are a lot of culture and historical implications to just the African-American culture as a whole, why people have a fear of health systems and doctors…”* (FG5-1, Black, Bisexual, 45, PLWH, Years since diagnosis not given). Similarly, a participant noted a tendency to be discounted by physicians, which could result in disengagement, *“…when you have doctors that don’t really listen to you, it’s hard to wanna go back and be seen or whatever. Some people just give up.”* (FG6-5, Black, Gay, 33. PLWH, 0-4.50 years since diagnosis). Such experiences could reduce opportunities for HIV testing, as this man explained,

*“Black men don’t go to doctors very regularly…We’re supposed to have an annual checkup every year…but before [I was diagnosed], I wouldn’t go see a doctor unless I was really, really sick…So a lot of people are not doing [HIV testing] because of what they’re afraid they’re gonna hear. They’re not doing it because we just don’t go to doctors.”* (FG5-13, Black, Gay, 56, PLWH, 0-4.50 years since diagnosis).

Similarly, a transgender participant noted that concerns about transphobia as a form of intersectional stigma in healthcare made testing more difficult for him as a GBQMSM,

*“…for trans people, even the idea of going to the doctor is really anxiety-provoking…knowing that you’re likely not going to be treated well and might have to extend a lot of the emotional health as well in order to get [HIV testing].”* (FG7-8, White, Queer, 26, HIV-negative).

By contrast, men noted that access to resources could moderate the frequency of negative experiences, and make it easier to have regular provider contact that facilitated HIV testing.

*“…socioeconomic status and privilege…I always benefited from extremely good health insurance with all the bells and whistles, and that hasn’t changed. And so even when I came out and started sleeping with guys, I knew that I would have exceedingly good health care. I knew that I could go to my doctor, and I wouldn’t be judged or mistreated…[that] has a tremendous impact in terms of whether or not you decide to get tested and make that a regular part of your lifestyle.”*(FG3-5, Asian, Gay, 22, HIV-negative).

“I think the bigger stigma is not getting tested nowadays”: Responsibility and regular testing.

Some participants acknowledged significant pro-testing norms among their associates—although they felt this might be limited to certain subgroups of GBMSM, such as those affiliated with the university or in a polyamorous community. Such participants described a pressure to test, with non-testing and irregular testing emerging as shameful, newly stigmatized behaviors. As these men said,

*“FG3-6: I think the bigger stigma is not getting tested nowadays…‘When was the last time you got tested?’ But we also live in a university town and things are different here…* (Asian, Gay, 21, HIV-negative).

*FG3-9: I see the same thing, but not just locally. I think on Reddit or other discussion boards, there’s definitely a norm that now people expect you will be getting tested regularly unless you’re in a monogamous relationship…* (White, Gay, 33, HIV-negative).

*FG3-8:…I think definitely there is that peer pressure to get tested in my circles, like, ‘Really? You haven’t yet? With the type of stuff you’re doing, you need to be going more than once a year, twice a year’…”* (White, Queer, 26, HIV-negative).

Participants noted the influence of HIV/AIDS-related public health and ASO outreach in developing these pro-testing norms. Specifically, they promulgated positive messages that could make GBQMSM feel good about testing, as one participant-test counselor stated. Noting the influence of such messages, a participant said,*“‘you get tested every six months.’ That was drilled in my head, whether you’re straight or gay, bi.’”* (FG1-8, Black, Gay, 45, PLWH, 4.51-10.99 years since diagnosis).

 Building on these messages, participants associated HIV testing—especially regular testing—with personal responsibility, praising testing as an expression of caring about one’s own health and that of others. Participants saw an emphasis on responsibility as an alternative to sexually stigmatizing messaging, *“I think the most important thing is, just frame [testing] in terms of being responsible and taking ownership of your health. So the whole idea of slut shaming doesn’t have to even come up.*” (FG7-7, White, Gay, 25, HIV-negative).

When discussing testing as a personal responsibility, GBQMSM participants characterized responsibility to test as having two dimensions: to one’s own health, and that of others. As this man said,

*“If you don’t get tested, I feel that’s just very shameful, in my eyes…That you don’t…care about your health or the health of others.”* (FG3-7, White, Bisexual, 31, PLWH, 0-4.50 years since diagnosis).

To participants, regular testing was clearly an issue of morality, *“The people that are getting tested, it’s like they genuinely care about others. And they care about themselves as well. Their morals are really up here.”* (FG4-4, Black, Gay, 31, PLWH, 22-31 years since diagnosis) Relatedly, GBQMSM characterized non-testing as willful ignorance maintained in order to avoid taking responsibility for stopping the spread of HIV. As these participants said,

*FG4-2: And it’s better, in their mind, it’s better to not know and then that way you don’t feel guilty if you go out and have unprotected sex…*(White, Gay, 49, HIV-negative).

*FG4-4: Yes. Oh my God.* (Black, Gay, 31, PLWH, 22-31 Years since diagnosis)

*FG4-1: Because then you don’t know if you’re passing it on or not.* (Latino, Bisexual, 45, HIV-negative)

This specifically became a flight from the responsibility to practice safer sex, according to another man, *“‘Out of sight, out of mind.’ Where if they don’t know, they can’t be responsible, ‘Well, I didn’t know I had it.’ But if you know that you had it and you gave it to somebody, that’s being irresponsible.”* (FG5-9, Black, Gay, 62, PLWH, 11-21.99 years since diagnosis) PLWH particularly chastised this behavior, as well as non-disclosure of HIV status to potential partners or those who did not take the possibility of spreading HIV seriously, contrasting their own behavioral commitments to such conduct, as this man stated,

*“…you have those out there that…pass it around…those are the people that I have an issue with because they make the rest of us look bad. And I’m not like that, me personally. That’s how I’ve always been. I care about everybody because I care about myself too much and I care about everyone else. So that just pisses me off.”* (FG4-4, Black, Gay, 31, PLWH, 22-31 years since diagnosis).

In terms of one’s own health, participants attributed a number of virtues to regular testers in addition to responsibility: maturity, realism, and positive self-esteem. As this man said,

*“The people that’s not getting tested, you can look at other aspects of their life and they seem real childish, they make stupid decisions and they don’t really take care of themselves…And they’re probably more self-aware, too.”* (FG5-6, Black, Gay, 31, PLWH, 11-21.99 years since diagnosis).

Additionally, positive self-esteem was linked to lack of internalized homophobia, with one man claiming that people who do not test regularly and proactively are those who, *“…won’t readily accept that they’re gay that have some self-loathing”* (FG9-5, White, Gay, 53, HIV-negative).

For some men, this moral pressure was linked to sharing testing behavior as a form of self-presentation as a “responsible person,” either in dialogue with other GBQMSM or online, *“I think some people will put on their profile, negative as of this recent data, look at me being so responsible. I think some people are driven by that.”* (FG7-7, White, Gay, 25, HIV-negative) Because of the moral value attached, regular testing could be a way to resist stigma associated with presumed sexual immorality,

*“I got a lot of…stigma from family and for me, getting regularly tested, and showing that it’s possible to be sexually active and still be responsible and take care of my health was a way of proving to my family that, no, you’re wrong.”* (FG3-5, Asian, Gay, 22, HIV-negative).

Notably, some HIV-positive participants criticized this moral pressure to test regularly for HIV, highlighting the importance of **being “ready”** for the personal impact of an HIV test—both mentally and socially. Without appropriate supports, they felt, delaying or avoiding testing was an important alternative. These men resisted what they perceived to be moral pressures around HIV testing, arguing that messages could be simplistic and fail to take the difficult realities of testing positive into account. As this man said,

*“People automatically assume, especially people in the healthcare field, especially people at the university… ‘Get tested! Get tested! It’s the right thing to do. It’s a no-brainer. Just get tested. It’s responsible and you should feel like crap if you don’t…However, there is a whole mess of baggage that comes with that, that you actually need to weigh the pros and cons of that very carefully…”* (FG2-4, Black, Gay, 37, PLWH, 11-21.99 years since diagnosis).

Participants highlighted the immense consequences of a positive diagnosis for their mental health, for example, *“…if it puts you in a state of being suicidal, it doesn’t solve anything. If a week difference or a year difference would circumvent you wanting to be suicidal…. just earlier the better? I don’t believe that, especially if you’re alone, you have no parents…”* (FG2-2, Black, Bisexual, 41, PLWH, 4.51-10.99 years since diagnosis). Similarly, another man described his experience of testing positive as deeply traumatic, arguing for the right to delay testing of one was not ready. He said,

*“I really wasn’t prepared for all that was about to happen to me…even being positive or not, if I just waited three years…I would have had three more years of freedom, three less years of stigma…and then I’ll deal with that when I’m on my deathbed.”* (FG2-3, Black, Gay, 22, PLWH, 0-4.50 years since diagnosis).

Without appropriate supports, these participants felt that delaying or avoiding testing was an important alternative to early testing. Thus, a downside of moral pressure to get an HIV test could be people learning of their HIV positive status when ill-equipped to deal with this new reality.

“*It just came along with the check-up*”:  HIV stigma and testing as routine care.

 In terms of linking HIV testing to personal health care, several participants discussed regular HIV testing as a form of routine care—not something shameful or a response to “bad” behavior. These statements typically compared HIV testing to healthcare for other, non-stigmatized conditions, as these men said,

*“You’re supposed to get a check up every six months to a year…if you get a check up, that should be something on the list. Cholesterol, blood pressure, HIV.”* (FG4-2, White, Gay, 49, HIV-negative).

*“Some of us…we’re raised with this as something that they were very conscious of, started getting tested before we started having sex, and it’s just part of life, and we’re used to it. Every six months, the same way that I get my dentist appointment, I get my test.”* (FG7-8, White, Queer, 26, HIV-negative).

For participants, framing HIV testing as routine care challenged the differential treatment inherent in stigma. More broadly, GBQMSM identified integration of HIV testing into other healthcare as a valuable stigma reduction strategy that might increase comfort with seeking testing. This approach was supported by healthcare providers—whom participants viewed as GBQMSM-competent—offered HIV testing as part of routine care,

*“When I looked at another city, I went to a specifically gay doctor practice [chuckle] and it didn’t matter if I wanted it or not, he was like, “You’re gonna get tested,“ it just came along with the check-up.”* (FG3-10, White, Gay, 33, HIV-negative).

More broadly, participants identified integration of HIV testing into other forms of healthcare as a stigma reduction strategy,

*“…they should try to avoid an organization meant for just …coming for [HIV] testing. It should be like, for example, in a hospital where people come for diabetes, people come for heart problems, they just do testing. So you can’t tell, because usually people are free to come in to a building like this…”* (FG1-6, White, Gay, 50, PLWH, Years since diagnosis not given).

## Discussion

Drawing from focus groups with GBQMSM in Metropolitan Detroit, we presented GBQMSMs’ perspectives regarding how stigma affects three HIV testing micro-decisions. With respect to *whether to get tested*, GBQMSM believed that stereotyping of HIV testers as sexually promiscuous could impede testing, especially when imagining negative responses of healthcare providers. These stereotypes also affected HIV risk perceptions; thus, they sometimes spawned appropriate testing, and sometimes resulted in test avoidance or inaccurate risk perceptions. Many participants, particularly younger men, also believed that fears of sexual rejection—especially rooted in stigmatizing messages including cleanliness metaphors on online hookup sites—could undermine decisions to test. However, some participants observed some advocacy against stigma among GBQMSM within these settings. Fear of rejection from friends and family could also prevent HIV testing, with “horror stories” shared by PLWH offering negative examples about what to expect. As for *where to get tested*, some Black participants expressed fears of being identified when seeking HIV testing, and of being labeled as “HIV-positive” and the target of gossip. This could lead to decisions to seek testing outside of one’s community. Furthermore, participants had experienced stigma concerning their sexual behaviors and identities from healthcare providers, which led them to search for specialized testing services that were GBQMSM-competent. In terms of *how often to get tested*, GBQMSM might get tested infrequently if they avoided healthcare in general because they experienced or feared discrimination such as racism and transphobia from healthcare providers. Testing frequency decisions were also affected by stigma towards non-testing, with regular testing seen as a moral behavior indicating care for oneself and others. Yet, some PLWH participants noted a downside to perceived moral pressure: the possibility of receiving an HIV-positive test without necessary supports. Finally, it was felt that treating HIV testing as the same as any other routine care could simultaneously reduce stigma and promote regular testing.

As in previous research [43, 68, 69], our participants posited that promiscuity stereotypes influenced their perceptions of HIV risk [70], resulting in concerns about being judged as promiscuous when seeking testing or if testing positive. GBQMSM with multiple partners are judged negatively, with the promiscuity stereotype underlying prejudice towards gay men [71–75]. Furthermore, GBQMSM themselves may endorse the stereotype that, as a group, they are promiscuous [76], which some study participants characterized as a form of self-stigma/internalized homophobia. Participants also noted that GBQMSM often judge one another’s sexual behavior, although some men had reclaimed labels such as “slut” or “ho.” Unfortunately, such judgement, sometimes called “slut shaming,” can impede GBQMSMs’ conversations about HIV risk [77] and reduce opportunities for social support surrounding HIV prevention and testing. Beyond the HIV context, this stereotype is also psychologically harmful to at least some GBQMSM: endorsement of self-stereotypes among GBQMSM, including those that GBQMSM are promiscuous, has been associated with symptoms of depression, anxiety, and stress [78]. With such negative impacts, there is a clear need for HIV testing interventions that challenge this stereotype. Given that promiscuity stereotypes have also plagued efforts to promote PrEP uptake [47, 48, 79], novel, pro-sex campaigns (e.g., [49]) could simultaneously address HIV testing and PrEP uptake. Such campaigns could leverage the diversity of values among GBQMSM, and as reflected in this study, by supporting men to hold community dialogues concerning promiscuity stereotypes, personal risk assessments, and self-stigma/internalized homophobia.

A novel finding was the extent to which participants stressed that fears of sexual rejection were a stigma-related concern undermining HIV testing. Although previous research has identified that GBQMSM living with HIV often worry about, and experience, sexual rejection [80–82], and HIV-negative men report avoiding sexual encounters with PLWH [83], we are unaware of previous work that links specific fears of sexual rejection to HIV test avoidance among HIV-negative GBQMSM. Moreover, although previous research has shown associations between anticipated stigma (expected rejection and discrimination) and HIV testing delays [5, 6], this work has not highlighted particular concerns about rejection in a sexual context. However, the negative impacts of sexual rejection in the lives of GBQMSM can perhaps be inferred from work showing that sexual rejection can negatively affect the self-esteem and confidence of GBQMSM living with HIV [81]—especially given that it has even been positively associated with suicide ideation and attempts in this group [84]. Taken together, our findings and other work on sexual rejection highlights a need to reduce the sexual stigmatization of PLWH, including as one step towards encouraging testing behavior among GBQMSM. Communication messages that warranted include those that clarify the ability to avoid HIV transmission through effective use of biomedical prevention options, including TasP (e.g., “Undetectable=Untransmittable” or “U=U” messages) and PrEP, and that affirm the sexual desirability of PLWH. Furthermore, given that younger men more commonly expressed this concern, it may be valuable to create such messages specifically targeted to young GBQMSM.

Furthermore, although GBQMSM living with HIV may prefer meeting partners online due to the ability to avoid rejection after investing in someone personally (e.g., [85]), participants clearly identified social networking and dating sites as a major site of enacted HIV/AIDS stigma. Several aspects of such sites may heighten stigmatization between GBQMSM, including the potentially greater frequency with which HIV disclosure may occur online as opposed to face-to-face interactions. Furthermore, stigma begins with labeling of differences [3]; yet, social networking and dating sites typically provide predetermined categories for building identities and connections [86], restricting GBQMSM to “menu-driven identities” [87]. Indeed, hookup apps for GBQMSM now provide menu-driven options to disclose use of biomedical HIV prevention, including PrEP and TasP [88, 89]. In addition to labeling, stereotyping may be more salient in online environments where people do not know one another and a lack of information about individual characteristics results in “de-individuation” [90]. This may amplify the effects of cues regarding category membership (e.g., HIV status), focusing attention on stereotypical characteristics [91–93]. Participants also described practices of distancing towards PLWH on such sites, such as ignoring them or posting a desire only for HIV-negative men. Given the foregoing, a potential approach to reducing HIV/AIDS-related stigma and its impacts on testing is to design social networking and dating platforms with a goal of disrupting HIV stigmatization. As we are pursuing in current work, stigma reduction may be possible through novel platform design choices that reduce perceptions of difference between HIV-positive and –negative GBQMSM [94]. Interventions may also profit from leveraging the activism of some GBQMSM against stigma [95], such as that documented in this study. By intervening in the environment in which stigmatization occurs, such interventions may address new socio-contextual factors on a pathway to promoting HIV testing uptake [96].

As in previous research [8], we found that stigma concerns vary based on HIV testing location. Across participants, a common dynamic was to seek specialized services for GBQMSM—often after negative experiences with Michigan healthcare providers. Of great concern is the fact that some GBQMSM experienced denial of care and urging to seek HIV prevention care elsewhere; this aligns with broader concerns about provider-based stigma [41]. It also resonates with a 2017 US survey that found that 8% of LGB people had a healthcare provider refuse to see them due to their sexual orientation in the past year [97]. In the face of potential or enacted discrimination from Michigan healthcare providers, participants deliberately sought GBQMSM-competent healthcare providers and specialized HIV/AIDS services. With respect to specialized services, this option is due to policy efforts to fund confidential or anonymous voluntary counseling and testing [98]. For healthcare needs beyond HIV testing, however, finding such LGBTQ-competent providers may not always be easy [99, 100], although telehealth may increase geographic access to such providers where available [101]. GBQMSM who are not “out” may use healthcare less after experiencing stigma and discrimination from healthcare providers [102]. To improve access to healthcare for GBQMSM, local initiatives include a list of LGBTQ-affirming healthcare providers [103], and a digital intervention, currently undergoing an efficacy trial, that aims to make GBQMSM aware of GBQMSM-competent providers by assessing them using a “secret shopper” methodology, and referring them to competent testing locations [104]. Furthermore, a new clinic dedicated to LGBTQ healthcare in Detroit [105] was recently launched; this approach, which expands the range of specialized services available, has elsewhere been shown to reduce perceptions of sexuality-related stigma [106] and to increase testing uptake among GBQMSM [107]. Nevertheless, there is a need to ensure that individuals who do not seek, or do not know about, specialized services, still receive non-discriminatory care. Thus findings reveal a need for greater policy attention to, and interventions for, stigma reduction and LGBTQ-competence among healthcare providers in Metropolitan Detroit [60, 108], and in the institutions that train them. For example, medical education should expand training of providers concerning care for GBQMSM and HIV screening [109].

With respect to frequency of HIV testing, findings revealed two sets of discourses and institutional practices that promoted regular testing. The first can be called a “responsibility discourse” whereby organizations and individuals promote the view that HIV testing is the “responsible thing to do.” The second can be termed a “routine care” discourse, in which proponents assert that testing should be a part of routine preventative care, similar to cancer screening. Participants in this study argued both positions, and described testing prompted by both views of frequent testing. With respect to the responsibility discourse, participants’ perspectives align with previous research showing a positive association between a desire to avoid transmitting the virus [110], and testing [7]. Some participants also described interactions in which they accepted testing when offered by their healthcare providers as routine care practices such as new patient visits and checkups. Similarly, a Canadian study found that routine testing lowered barriers by suggesting that “anyone could benefit” from learning their HIV status [111]. Notably, participants felt that this approach inherently reduced stigma by treating HIV as similar to other health concerns. Indeed, providers who support routine HIV testing may assert that HIV is “no different” from other conditions [112], and normalize HIV testing by drawing parallels to other preventative care [113]. Experiments also demonstrate that using opt-out rather than opt-in testing can decrease perceived stigma by communicating that testing is the norm, increasing rates of testing uptake [114].

 Although both “responsibility” and “routine” discourses were endorsed by participants, and may have encouraged their HIV testing, we must note differences in their reception across different GBQMSM subgroups. Specifically, participants highlighted a differential, and negative, impact of framing HIV testing as a personal responsibility on men with undiagnosed HIV infection. In line with public health interventions that communicate moral imperatives to reduce health risks [115, 116], participants described the creation of a “new stigma” towards non-testing. In the face of this perceived “new stigma,” some PLWH participants stressed that the pressure to get tested could lead to testing without proper support, leading to emotional distress that they were ill-equipped to manage. Others have highlighted an emerging moral pressure to seek HIV testing, noting that this pressure fails to acknowledge the nature of HIV/AIDS as a syndemic linked to discrimination, substance use, and mental health concerns [117, 118]. As an alternative, study participants advocated the right to be “ready” before testing, contending that HIV testing interventions should include more nuanced discussions that acknowledge possible negative consequences of testing, and help to build “readiness” for people with undiagnosed infection. Building on participants’ perspectives and extant literature [119–122], we argue that such interventions could profitably focus on addressing poverty or homelessness, building social support networks and coping skills, providing substance use disorder treatment as applicable, and preparation for linkage to HIV care in the event of a positive diagnosis. In contrast, participants did not express concerns about the impact of the “routine care” discourse, suggesting that this approach to message framing may be preferable since it appears to avoid the pitfalls of creating a “new stigma.”

Finally, extending prior work on unique stigma experiences among Black GBQMSM due to intersectionality [123], we highlight several stigma-related issues that Black GBQMSM experienced to a greater extent than participants from other racial/ethnic groups. In this study, these include fear of family rejection, fear of being identified at HIV testing sites and presumed to be living with HIV, and general avoidance of healthcare—in part due to fears of, or experienced, racism. This suggests ways in which stigma may differentially affect Black GBQMSM, subsequently affecting their HIV testing decisions. With respect to fears of family rejection, the relative strength of this concern may be based in personal experiences or those of known others, and rooted in participants’ family and religious values and related moral judgements (e.g., [124])—especially given that Detroit is an area in which Black churches have a history of opposing homosexuality [125, 126]. In turn, such perspectives have the potential to influence congregants’ attitudes (e.g., [127]), who would likely include some of their own family members. As for where to get tested, a new finding was the extent to which Black GBQMSM worry that HIV testers, like PrEP users [46], may be labeled as “HIV-positive” by others, which may result in gossip that testers seek to avoid. Notably, this dynamic has only been mentioned briefly in previous work—in this case with racial minority groups in Australia [9]. For our participants, this resulted in choices to avoid being seen entering a building and sitting in a waiting room linked to HIV testing. In participants’ accounts, the availability of multiple testing site options, including outside of one’s community, provided greater privacy assurances. In terms of testing frequency, study results also showed that, as in previous work [19, 128], perceived stigma and discrimination from healthcare providers related to GBQMSM and other stigmatized identities such as race drove GBQMSM to specialized services in some cases and impeded testing in others cases—partly by reducing contact with providers [129]. While in need of confirmation via quantitative research, these findings suggest a need to target HIV testing interventions to the particular stigma-related concerns of Black GBQMSM. Such interventions could focus on building family support and acceptance for GBQMSM of all ages regardless of HIV status (e.g., [130]), ensuring awareness of multiple testing options including home testing [131, 132] for Black GBQMSM who live in smaller communities (and for rural-dwelling GBQMSM). Home testing could also be helpful for addressing concerns about facing stigma in health care. Finally, integrating LGBTQ+-related concerns into broader efforts to enhance racial justice in healthcare [133].

### Limitations

This study has limitations. First, because we focused on GBQMSM in Metropolitan Detroit, the experiences we identified may not be identical to those of other communities. While we recruited a diverse sample of GBQMSM, a survey would be useful for identifying the prevalence of the surfaced themes. Given that we used face-to-face focus groups, some individuals who experience stigma about their sexual behavior, testing behavior, or HIV status may have chosen not to participate. A study using an anonymous design may enable participation of such GBQMSM. The majority of GBQMSM in our sample (89.1%) had received an HIV test, suggesting that those GBQMSM who may have never received a test due to stigma may be underrepresented in our study. We also did not specifically probe about biphobia; as such, there could be undiscovered differences between bisexual or queer men and participants concerning their experiences of stigma. Furthermore, we did not ask about participants’ degree of “outness” about their sexuality or HIV status; this may have affected participants’ perceptions of stigma, especially in interactions with healthcare providers and family members.

## Conclusions

According to GBQMSM, decisions about *whether to get tested* was affected by stigma through stereotyping of HIV testers as sexually promiscuous, and through fears of sexual rejection if they tested positive. Fear of rejection from friends and family, rooted in others’ “horror stories,” could also prevent HIV testing. Stigma concerns played a role in decisions about *where to get tested*, with some Black participants fearing being identified when seeking HIV testing, and assumed to be PLWH. Furthermore, many participants had experienced sexuality-related stigma from healthcare providers, which led them to search for GBQMSM-competent testing services. GBQMSM might get tested infrequently if they avoided healthcare due to enacted and/or anticipated racism and transphobia from healthcare providers. Testing frequency decisions were also affected by stigma towards non-testing, and treating HIV testing as the same as any other routine healthcare. Results can help to inform future interventions designed to promote HIV testing by reducing the stigma and discrimination that diverse GBQMSM face.

## Supplementary Information

**Additional file 1.**

## Data Availability

The qualitative data gathering during this study is not publicly available and cannot be shared due to confidentiality, since participants are potentially identifiable from the information contained in the data. Furthermore, ethical restrictions imposed by the consent form signed by participants prevents data sharing and data requests. Any questions about this can be directed to the corresponding author.

## Abbreviations

GBQMSM: Gay, bisexual, queer and other men who have sex with men
PrEP: Pre-exposure prophylaxis
TasP: Treatment as prevention
