# Supplementary material for: The impact of stigma on HIV testing decisions for gay, bisexual, queer and other men who have sex with men: a qualitative study
Source: BMC Public Health. 2022 Mar 9;22:471. doi: 10.1186/s12889-022-12761-5 (PMC8908600; doi:10.1186/s12889-022-12761-5)
Supplement: Supplementary file 1 — Additional file 1. [file 12889_2022_12761_MOESM1_ESM.docx]

**Developing a Stigma Reduction Intervention to Increase Uptake of HIV Testing and Prevention among MSM**

**Focus Group Discussion Guide**

*[Note: in keeping with the principles of qualitative research, these questions may evolve through the course of this research. However, this interview guide shows the possible range of questions to be asked.]*

**Introductory Statement:** Before we start, I’d like to thank you all again for agreeing to participate in this study. Your participation will contribute to better understanding of issues relevant to the sexual health of men who have sex with men. To protect your privacy and the privacy of other people in your life, please use only a first name, initial, or alias when talking about these people. I’d like to remind you that this conversation is confidential, so feel free to speak candidly. The idea is not to come to an agreement on answers to these questions, but to hear a diverse set of thoughts and opinions – there are no right or wrong answers. In order to promote discussion, we encourage everyone to be respectful and polite.

**PART A: Communities and Social Networks (30 min)**

1. What does the word “community” mean to you?

Probes:

- Do you think of yourself as having a “community”? If so, who is a part of it?
- Is there anyone that you think is NOT part of your community? If so, who?
- When do you interact with members of your community?
- How do you interact with members of your community?
- Where do you interact with members of your community?
  - How much do you interact with members of your community online or on social media? If so, which sites, and how do you interact on them?

1. If you think about all of the men who have sex with other men in southeastern Michigan, what different subgroups are there?

Probes:

- Where do members of these subgroups live?
- How different are these subgroups from one another in terms of the ages of their members?
- How different are these subgroups from one another in terms of race of their members?
- How much are transgender women or men a part of these subgroups?

1. How much do HIV-positive and HIV-negative MSM in southeastern Michigan interact with each other?

Probes

- When do HIV-positive and HIV-negative MSM interact?
- Where do HIV-positive and HIV-negative MSM interact?
  - How much do they interact online or on social media? If so, which sites, and how do they interact on them?
- Are HIV-positive people more likely to be part of any of the subgroups you talked about?
- Are HIV-negative people more likely to be part of any of the subgroups you talked about?
- Why do you think HIV-positive and –negative people interact as much as they do, and in the ways that they do?

1. Imagine that you are speaking with a gay man who has just moved to southeastern Michigan. He wants to meet lots of different people and make new friends. What would be the best way for him to find out about the different subgroups that you were talking about?

Probes:

- What would be the best way for him to connect with ___ subgroup?
- What would be the best way for him to connect with ___ subgroup?

1. Some people are good at connecting with lots of different people and groups. For example, a man might part of a group of Basketball players, a community of students at a particular university, and attend a specific Church. When a person is in that kind of position, they link different subgroups, and we call them a “network bridge”. If I wanted to find people that connect different subgroups of MSM, or network bridges, what would be the best way to do so?

Probes

- How easy or hard is it to know if a person is a network bridge?
- Is there any place I should visit?
- How would I identify a person like this at that place?
- Does this person do anything in particular to connect those different groups?

Examples: Share information, hold parties, introduce people to one another…

- How much should I use social media or the Internet to find this out?
  - If yes, which sites, and how should I use them?

**PART B: Stigma and HIV testing (30 min)**

1. Who do you think **should** get tested for HIV?
2. Who do you think **does** get tested for HIV?

Probes:

- How much have other people told you that they had been tested?
- Who has told you that? What did they say about it?

1. Do you think there is a difference between those who should get tested and those who do get tested? If so, why do you think there is that difference?
2. It is recognized that HIV/AIDS is a stigmatized disease. Stigma involves negative labels and stereotypes about people with HIV/AIDS, people distancing themselves from people with HIV/AIDS, and a loss of status for people with the disease. Do you think stigma has an impact on MSMs’ decisions to get tested for HIV? If so, what is the impact?

Probes:

- How much have other people told you that stigma affected their testing decisions?
- Who has told you that? What did they say about it?
- Has it ever affected your own decisions regarding HIV testing?

1. Pre-exposure Prophylaxis, or PrEP, is a daily pill that people who do not have HIV can use to prevent HIV infection. Where have you learned about PrEP so far? What have you learned about PrEP?
2. Who do you think **should** be on PrEP?
3. Who do you think **is** on PrEP?

Probes:

- Do you know anyone who is on PrEP?
- What do you know about their decision to get on PrEP?

1. Do you think there is a difference between those who should be on PrEP and those who are on PrEP? If so, why do you think there is that difference?
2. Do you think stigma has an impact on MSMs’ decisions to get on PrEP? If so, what is the impact?
3. If a group of community leaders wanted to reduce the stigma associated with testing and PrEP, what kinds of things should they deal with, and what specifically would you like them to do about it?

**Activity: Rank ideas**

**PART C: Network bridging and stigma reduction intervention (30 min)**

There have been a lot of programs outside of the HIV/AIDS area that try to reduce the stigma associated with different health conditions, and we are trying to figure out which ideas might work in an HIV/AIDS context in southeastern Michigan. So now we’re going to ask you for your opinions about several different program ideas.

1. What do you think about the possibility of a volunteer MSM who is openly HIV-positive working with a HIV-negative or status unknown MSM to help them get connected to HIV testing and/or PrEP? This might involve the HIV-positive MSM helping an HIV-negative or status-unknown MSM make an appointment, helping them get to an appointment, and/or attending the appointment with them. People probably wouldn’t know each other before the program. How much do you think MSM would participate in a program like this?

Probes:

- Why do you think they would participate that much?
- What do you think might be attractive about a program like that?
- What do you think might make them not want to participate in a program, like that?
- What impact, if any, do you think such a program would have on stigma?

*For HIV-positive focus groups only:*

1. Is there anything that could make a program like this more appealing for HIV-positive MSM?

*For HIV-negative focus groups only:*

1. Is there anything that could make like this more appealing for HIV-negative MSM or MSM who don’t know their HIV status?
2. Now imagine that the program focused on helping HIV-negative MSM get connected to HIV testing and/or PrEP was delivered by a volunteer network bridge like we talked about before? As a reminder, a network bridge is a lay person who connects different subgroups in southeastern Michigan. This volunteer network bridge would be of unknown HIV status. People might know each other before the program. How much do you think MSM would participate in a program like this?

Probes:

- Why do you think they would participate that much?
- What do you think might be attractive about a program like that?
- What do you think might make them not want to participate in a program, like that?
- What impact, if any, do you think such a program would have on stigma?

1. Now imagine that the program focused on helping HIV-negative MSM get connected to HIV testing and/or PrEP was delivered by a paid staff person like a case manager, early intervention specialist or community health worker. This paid staff person would be of unknown HIV status. People probably wouldn’t know each other before the program. How much do you think MSM would participate in a program like this?

Probes:

- Why do you think they would participate that much?
- What do you think might be attractive about a program like that?
- What do you think might make them not want to participate in a program, like that?
- What impact, if any, do you think such a program would have on stigma?

1. Is there anything else that we haven’t asked you about that you want to tell us about MSMs’ access to HIV testing and/or PrEP?

**Individual Survey**

*HIV-Related Care & Stigma*

***These questions give us some background information about you.***

1. **What is your birth month? __________ What is your birth year? __________**
2. **What is your gender?**

- Male
- Female
- Other (specify): ___________________

1. **What is your race? (Check all that apply)**

- Black or African American
- White
- American Indian or Alaskan Native
- Asian
- Native Hawaiian or Pacific Islander
- Other__________________________

1. **Are you Spanish or Hispanic or Latino?**

- Yes
- No

1. **Are you of Arab Descent?**

- Yes
- No

1. **What is your postal zip code? ___________________**
2. **Have you ever had any kind of sex with other men?**

- Yes
- No

1. **What is your sexual orientation?**

- Gay/homosexual
- Bisexual
- Straight/heterosexual
- Other (specify): ___________________

1. **What is the highest degree or level of school that you have completed? (check one)**

- Grade 8 or less
- Grades 9 to 12, no diploma
- High school graduate or equivalent (GED)
- Some college
- Associate degree (e.g., AA, AS)
- Bachelor’s degree (e.g., BA, BS)
- Graduate degree (e.g., MA, MS)
- Professional degree (e.g., MD, JD)

1. **What is your current employment activity? (check all that apply)**

- Working full-time (30 or more hours)
- Working part-time (less than 30 hours)
- Full-time student (12 or more credits)
- Part-time student (less than 12 credits)
- Unemployed
- Other: _______________________

1. **If you are working, what is your current job? ___________________________**
2. **What is your monthly income?**

- $0 - $1,000
- $1,001 - $2,000
- $2,001 - $3,000
- $3,001 - $4,000
- $4,001 - $5,000
- $5,001+

1. **How did you hear about this study? (check all that apply)**

- Advertisement on Grindr
- Advertisement on Scruff
- Advertisement on Facebook
- A friend’s social media post (e.g., on Facebook, Twitter or Instagram). Name of friend:______________________________________________________________________________
- An organization’s social media post (e.g., Spectrum Center, Unified or Necto’s posts on Facebook, Twitter or Instagram). Name of organization:_______________________________________________
- Post in Facebook Group. Name of Facebook group: ___________________________________________________________________________________
- Student email list. Name of email list: _____________________________________________________
- Organizational email list (e.g., Unified, Spectrum Center)
- Person approached me at a gay bar/event (e.g., at Necto)
- Flyer/card displayed in a gay bar. Name of bar:_________________________________________
- Flyer/card displayed at another type of business (e.g., coffee shop, restaurant, book store). Name of business:___________________________________________________________________________
- Flyer/ card displayed at a nonprofit or public organization (e.g., community center, recreation center, community college, university). Name of organization:__________________________________
- Organizational Newsletter (e.g., Spectrum Center, Unified, Affirmations). Name of organization: _____________________________________________________________________________________
- A staff member/volunteer at Unified, Spectrum Center or the University of Michigan contacted me directly (may be by phone, email, text, Facebook/Twitter private message, or face-to-face).

Name of staff member/volunteer (if known):________________________________________________

- A staff member/volunteer at Unified, Spectrum Center or the University of Michigan gave me a flyer or card. Name of staff member/volunteer (if known):_______________________________________
- A friend who is not affiliated with Unified, Spectrum Center or the University of Michigan approached me (by phone, email, text, or Facebook/Twitter private message, or face-to-face).
- Other: _______________________________________________________________________________

____________________________________________________________________________________________________________________________________________________________

*Now we will ask you some questions regarding things you do, and your opinions about them.*

| *I believe that…* | **Strongly Disagree** | **Disagree** | **Neutral** | **Agree** | **Strongly Agree** |
| --- | --- | --- | --- | --- | --- |
| 1. **For people who are HIV negative or don’t know their HIV status, getting an HIV test is the responsible thing to do.** | ❑ | ❑ | ❑ | ❑ | ❑ |
| 1. **For people who are HIV negative, going on Pre-Exposure Prophylaxis (PrEP) is the responsible thing to do.**   *PrEP (Pre-Exposure Prophylaxis) is an anti-HIV medication that can keep HIV negative people from becoming infected.* | ❑ | ❑ | ❑ | ❑ | ❑ |
| 1. **For people who are HIV positive, taking medications regularly is the responsible thing to do.**   *People who take medications regularly are less likely to transmit the virus to others.* | ❑ | ❑ | ❑ | ❑ | ❑ |

1. **How often do you talk about HIV/AIDS and related topics in all settings (e.g., home, work, with friends, online, offline, at parties, with partners, etc.)?**

- Never
- Rarely
- Not often
- Fairly often
- Very often
- Don’t know

1. **How often do you talk about HIV/AIDS and related topics online only (e.g., on Facebook, Twitter, online communities, Instagram, email. etc.)?**

- Never
- Rarely
- Not often
- Fairly often
- Very often
- Don’t know

1. **How often do you use each of the following social media sites?**

|  | **Circle One Answer** | | | | | | |
| --- | --- | --- | --- | --- | --- | --- | --- |
|  | **Never** | **Once a month or less** | **2-3 times a month** | **About once a week** | **2-6 times a week** | **About once a day** | **More than once a day** |
| Facebook | 1 | 2 | 3 | 4 | 5 | 6 | 7 |
| YouTube | 1 | 2 | 3 | 4 | 5 | 6 | 7 |
| Twitter | 1 | 2 | 3 | 4 | 5 | 6 | 7 |
| Google Plus | 1 | 2 | 3 | 4 | 5 | 6 | 7 |
| Instagram | 1 | 2 | 3 | 4 | 5 | 6 | 7 |
| Snapchat | 1 | 2 | 3 | 4 | 5 | 6 | 7 |
| Reddit | 1 | 2 | 3 | 4 | 5 | 6 | 7 |
| Tumblr | 1 | 2 | 3 | 4 | 5 | 6 | 7 |
| Pinterest | 1 | 2 | 3 | 4 | 5 | 6 | 7 |
| Vine | 1 | 2 | 3 | 4 | 5 | 6 | 7 |
| LinkedIn | 1 | 2 | 3 | 4 | 5 | 6 | 7 |
| Other______________________________________________ | 1 | 2 | 3 | 4 | 5 | 6 | 7 |
| Other______________________________________________ | 1 | 2 | 3 | 4 | 5 | 6 | 7 |
| Other______________________________________________ | 1 | 2 | 3 | 4 | 5 | 6 | 7 |

1. **Have you ever disclosed your HIV status on any of the above social media sites?**

- Yes
- No

1. **People have different definitions for the term “community”. Thinking about the different communities that you belong to, please indicate below what is the community that you feel like you belong the most:**

____________________________________________________

*Think about two different groups of people in your life – those you feel VERY CLOSE to, such as close family and friends, and those you feel SOMEWHAT CLOSE to. We’d like to know how many people in your life fit into each one of these categories.*

1. **Think about the people you feel VERY CLOSE to. This includes those you discuss important matters with, regularly keep in touch with fact-to-face, by phone, e-mail or other means, or are there for you when you need help. You should only include each person once. Thinking about ALL the people who are very close to you, how many are… ?**

*(Please list each person once only)*

- Main Partner(s) ___________________
- Casual Partner(s) ___________________
- Former Partner(s) ___________________
- Members of your immediate family: parents, siblings, adult children, or in-laws ___________________
- Other relatives ___________________
- People you know from work ___________________
- Neighbors ___________________
- Other people ___________________
- None

**TOTAL** number of people who you feel VERY CLOSE TO ___________________

How many of these people are women? ___________________

How many of these people are the same race as you are? ___________________

How many of these people are the same ethnicity as you are? ___________________

How many of these people are men who have sex with other men? ___________________

How many of these people are within 5 years of your age (older or younger) ___________________

1. **Now think about the people in your life who you feel SOMEWHAT CLOSE to. They’re more than just casual acquaintances, but they’re not as close as the friends and relatives in the previous question. Please mention each person only once. Thinking about ALL the people who are somewhat close to you, how many are… ?**

*(Please list each person once only)*

- Main Partner(s) ___________________
- Casual Partner(s) ___________________
- Former Partner(s) ___________________
- Members of your immediate family: parents, siblings, adult children, or in-laws ___________________
- Other relatives ___________________
- People you know from work ___________________
- Neighbors ___________________
- Other people ___________________
- None

**TOTAL** number of people who you feel SOMEWHAT CLOSE to ___________________

How many of these people are women? ___________________

How many of these people are the same race as you are? ___________________

How many of these people are the same ethnicity as you are? ___________________

How many of these people are men who have sex with other men? ___________________

How many of these people are within 5 years of your age (older or younger) ___________________

1. **Of the people/person you feel VERY CLOSE to AND the people/person you feel SOMEWHAT close to, how many know one another? Would you say…**

- They ALL know each other
- MOST of them know each other
- About HALF know each other
- Only SOME know each other
- NONE know each other
- Don’t know

1. **Some people are good at connecting with lots of different people and groups. For example, a man might part of a group of Basketball players, a community of students at a particular university, and attend a specific Church. When a person is in that kind of position, they link different subgroups, and we call them a “network bridge”. Can you think of anyone who you know who is in that kind of a position?**

- No (Skip to question 26)
- Yes

**25a. (If YES for question 25) What are their initials? _______**

**25c. (If YES for question 25) Are they an MSM (a man who has sex with other men)? _______**

**25f. (If YES for question 25) What subgroups does this person connect?**

**1.__________________________**

**2. __________________________**

**3. __________________________**

**4. __________________________**

**5. __________________________**

*Now we will ask you some questions your use of HIV testing and prevention services.*

1. **Have you ever been tested for HIV?**

- Yes
- No (Skip to question 26g)

**26a. (If YES for question 26) When did you last get tested?**

| _____________ | _____________ |
| --- | --- |
| Month | Year |

**26b. (If YES for question 26) The LAST time you got tested for HIV, did you pick up your results?**

- Yes
- No

**26c. (If YES for question 26) How many times have you gotten tested for HIV? ____________** times

**26d. (If YES for question 26) Out of all the times you have been tested for HIV, how many times did you pick up your results?** ____________ times

**26e. (If YES for question 26) The LAST time you got tested for HIV, why did you do so? (Indicate all that apply)**

- Had symptoms that I thought were HIV
- Had an STI
- Condom broke
- Started a new relationship
- I get tested regularly
- To set an example for others
- I thought I was at risk
- I was encouraged to get tested by a partner
- I was encouraged to get tested by a friend or family member
- I was encouraged to get tested by a healthcare provider
- I found information online that made me think I was at risk
- Other (specify): ___________________

**26f. (If YES for question 26) The LAST time you got tested for HIV, where did you do so?**

- Hospital
- Primary Care Clinic/Physician
- AIDS service organization (e.g., Michigan Unified, Lansing Area AIDS Network)
- Home test
- Public Health Department
- Planned Parenthood
- Other (specify): ___________________

**26g. (If NO for question 26) What are your reasons for not getting tested? (Indicate all that apply)**

- Fear of finding out the results
- Cost
- Unaware of where to get tested
- Concern that my sexual partners may be contacted
- I’m not at risk of getting HIV
- Afraid the test would hurt
- Shame or embarrassment
- Even though I haven’t tested for HIV, I know that I’m HIV positive
- Other (specify): ______________________________________________________________

1. **Have you ever used PrEP?**

*PrEP (Pre-Exposure Prophylaxis) is an anti-HIV medication that can keep HIV negative people from becoming infected.*

- Yes
- No (Skip to question 27d)

**27a. (If YES for question 27) When did you start PrEP?**

| ________ | ________ |
| --- | --- |
| Month | Year |

**27b. (If YES for question 27) Why did you start PrEP? (Check all that apply)**

- Had an STI
- Had/have a partner who has HIV
- To set an example for others
- I thought I was at risk
- I was encouraged to start taking PrEP by a partner
- I was encouraged to start taking PrEP by a friend or family member
- I was encouraged to start taking PrEP by a healthcare provider
- Other (specify): ______________________________________________________________

**27c. (If YES for question 27) Did you stop taking PrEP? If so, when?**

- Yes
- No

| ________ | ________ |
| --- | --- |
| Month | Year |

*(Leave blank if you did not stop taking PrEP)*

**27d. (If NO for question 27) What are your reasons for not starting PrEP? (Indicate all that apply)**

- I am HIV-positive
- I didn’t know about PrEP / I didn’t know what PrEP was
- Cost (too expensive)
- I’m not at risk
- Afraid of side effects
- Shame or embarrassment
- I don’t know where or how to get PrEP
- Not covered by my health insurance
- Other (specify): ___________________

1. **Have you ever tested positive for HIV?**

- Yes
- No (skip to question 28c)

**28a. (If YES for question 28) When did you test positive?**

| ________ | ________ |
| --- | --- |
| Month | Year |

**28b. (If YES for question 28) Are you currently on HAART (Highly Active Antiretroviral Therapy)?**

- Yes
- No

**28c. (If NO for question 28) How easy or hard would it be for you to find a location nearby to get an HIV test? Please rate on a scale of 1-10 (1 being harder, 10 being easier) _____________**

**THANK YOU!**
